# Supplementary material for: Clinicopathological Features of Small Pancreatic Neuroendocrine Neoplasms 10 mm or Smaller
Source: Diagnostics (Basel). 2025 Sep 23;15(19):2423. doi: 10.3390/diagnostics15192423 (PMC12523643; doi:10.3390/diagnostics15192423)
Supplement: Supplementary file 1 [file diagnostics-15-02423-s001.zip › diagnostics-3839851-supplementary/Supplementary files/Supplementary Table S1.pdf]

Supplementary Table S1. Comparison of imaging findings between PNENs ≤10 mm and >10 mm excluding functional tumors and MEN1 cases

	≤10 mm (n = 31)	>10 mm (n = 45)	*P*-value
Images			
Typical	22 (71.0)	8 (17.8)	< 0.001
Atypical	9 (29.0)	37 (82.2)	
Shape			
Regular	29 (93.6)	26 (57.8)	< 0.001
Irregular	2 (6.5)	19 (42.2)	
Internal uniformity			
Homogeneous	30 (96.8)	21 (46.7)	< 0.001
Heterogeneous	1 (3.2)	24 (53.3)	
Early enhancement			
Present	27 (87.1)	24 (53.3)	0.003
Absent	4 (12.9)	21 (46.7)	
Calcification			
Absent	30 (96.7)	34 (75.6)	0.022
Present	1 (3.3)	11 (24.4)	
Cystic degeneration			
Absent	29 (93.6)	37 (82.2)	0.185
Present	2 (6.4)	8 (17.8)	
MPD dilation			
Absent	31 (100)	31 (68.9)	< 0.001
Present	0 (0)	14 (31.1)	

Data are expressed as number (percentage).

Abbreviations: PNEN, pancreatic neuroendocrine neoplasm; MEN, multiple endocrine neoplasia; MPD, main pancreatic duct
