# Supplementary figures and images for: Clinicopathological Features of Small Pancreatic Neuroendocrine Neoplasms 10 mm or Smaller
Source: Diagnostics (Basel). 2025 Sep 23;15(19):2423. doi: 10.3390/diagnostics15192423 (PMC12523643; doi:10.3390/diagnostics15192423)

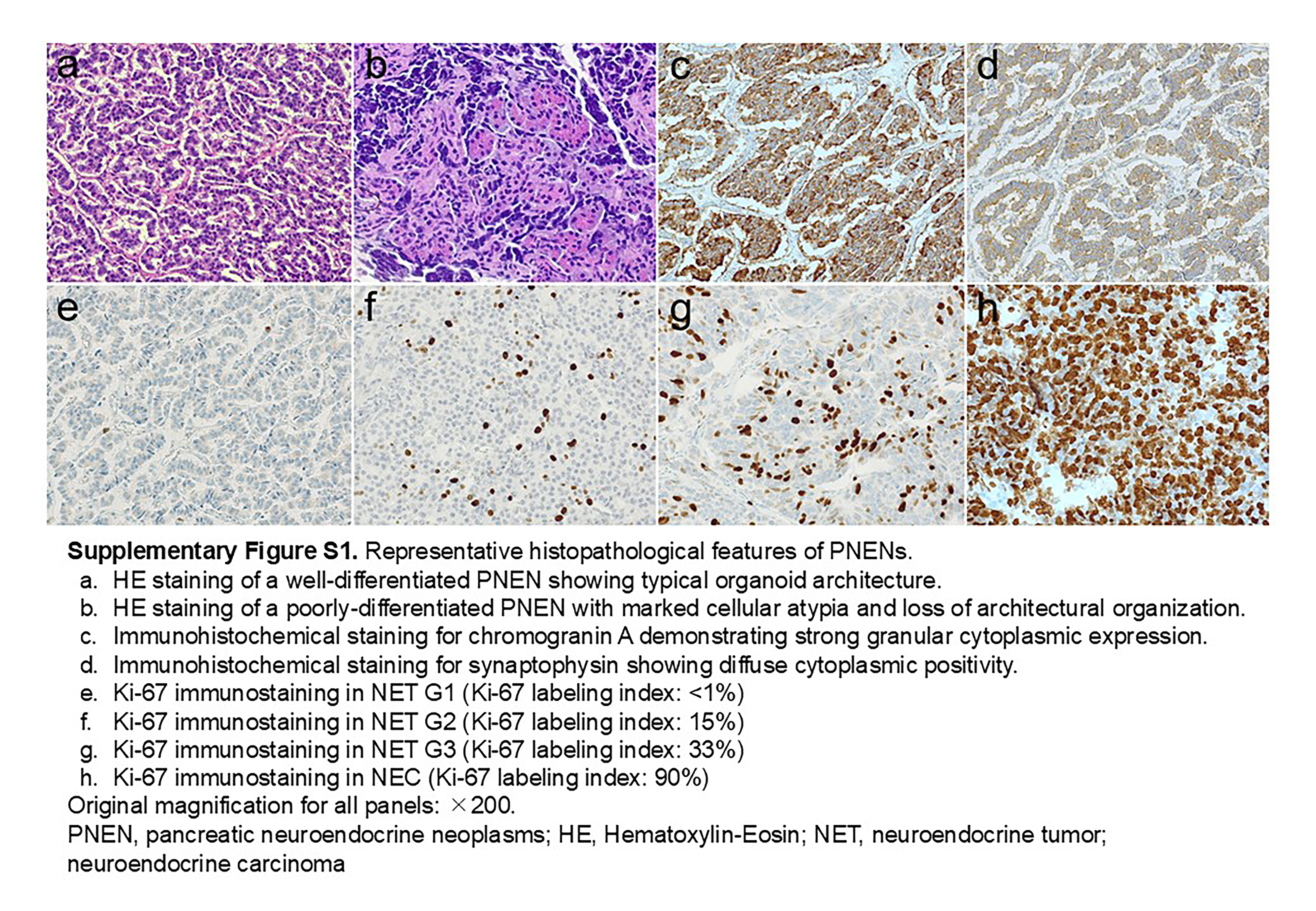

Supplement: Supplementary file 1 [file diagnostics-15-02423-s001.zip › diagnostics-3839851-supplementary/Supplementary files/Supplementary Figure S1.jpg]

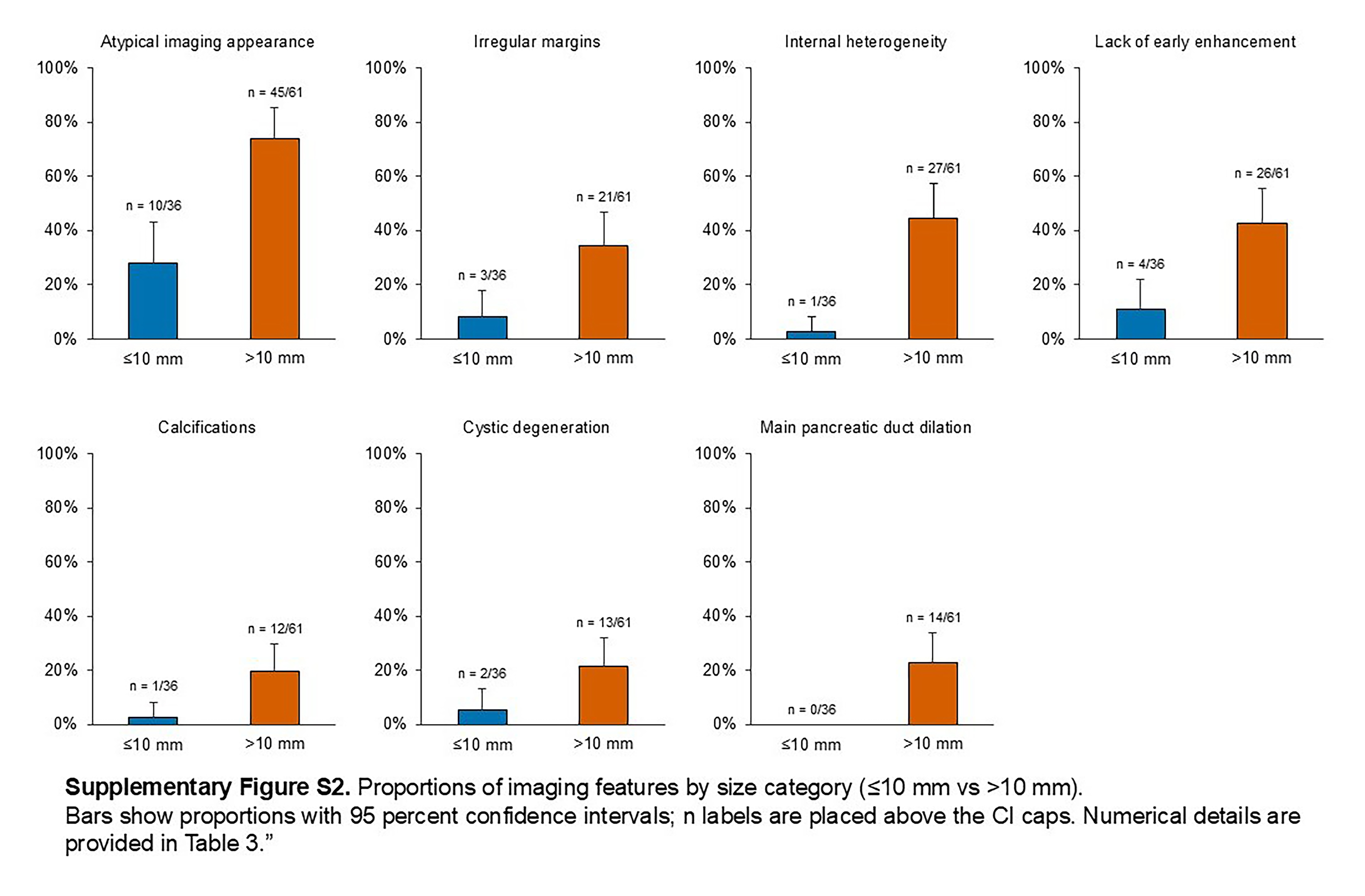

Supplement: Supplementary file 1 [file diagnostics-15-02423-s001.zip › diagnostics-3839851-supplementary/Supplementary files/Supplementary Figure S2.jpg]
